# 

**Published:** 1795

**Authors:** 


*Catalogue of Books.* — — 223

*Index.* — — — 228

---

DIRECTIONS TO THE BINDER.

Plate the Firſt, the references to which are explained in pages 92 and 116, may be placed at page 92; and plate the ſecond at page 121.

### CATALOGUE OF BOOKS.

1. THOUGHTS on the Effects of the Application and Abstraction of Stimuli on the Human Body; with a particular View to explain the Nature and Cure of Typhus. By *J. Wood*, M. D. 8vo. *Murray*, London, 1793.

2. An Account of the Bilious, Remitting, Yellow Fever, as it appeared in the City of Philadelphia in the year 1793. By *Benjamin Rush*, M. D. 8vo. Philadelphia, 1794.

3. Observations on the Cause, Nature, and Treatment of the Epidemic Disorder prevalent in Philadelphia. By *D. Nassy*, M. D. Member of the American Philosophical Society. 8vo. Philadelphia, 1793.

4. A Short Account of the Malignant Fever, lately prevalent in Philadelphia; with a Statement of the Proceedings that took place on the Subject in different Parts of the United States. By *Matthew Carey*. 8vo. Philadelphia, 1793.

5. A Treatise on the Extraction of the Cataract. By *Frederick Bischoff*, F. M. S. Oculist to his Majesty in the Electorate of Hanover,

 and and to her Majefty in England. 8vo. *Nicol*, London, 1793.

6. An Account of a Fever which appeared in feveral Parts of Somerfetfhire in the year 1792. By *Richard Poole*, Surgeon, Sherborne. 8vo. *Johnfon*, London, 1793.

7. A Guide for Self-Prefervation and Parental Affection; or plain Directions for enabling People to keep themfelves and their Children free from feveral common Diforders. By *Thomas Beddoes*, M. D. 12mo. *Murray*, London, 1793.

8. A Chemical Differtation on the Thermal Waters of Pifa, and on the neighbouring acidulous Spring of Afciano; with an Hiftorical Sketch of Pifa, and a Meteorological Account of its Weather. To which are added, Analytical Papers refpecting the Sulphureous Water of Yverdun. By *John Nott*, M. D. of Briftol Hot-wells. 8vo. *Walter*, London, 1793.

9. Horti Botanici Cantabrigienfis Catalogus. 8vo. Cantabrigiæ, 1794.

10. Flora Oxonienfis, exhibens Plantas in agro Oxonienfi fponte crefcentes, fecundum Syftema fexuale diftributas. Auctore *Joanne Sibthorp*, M. D. Profeffore Regio Botanico, Regiæ Societatis Londinenfis aliarumque Societatum Socio. 8vo. Oxonii, 1794.

11. Differ-

11. Dissertatio Inauguralis de Angina maligna. Auctore *Arthuro Bedford*, Anglo. 8vo. Edinburgi, 1792.

12. Dissertatio Inauguralis de Respiratione. Auctore *Thoma Blair*, Scoto-Britanno. 8vo. Edin. 1792.

13. Dissertatio Inauguralis de Variolis. Auctore *Joanne Bower*, Scoto. 8vo. Edin. 1792.

14. Dissertatio Inauguralis de Visu. Auctore *Wheaton Bradish*, Hiberno. 8vo. Edin. 1792.

15. Dissertatio Inauguralis de Rheumatismo acuto. Auctore *Joanne Bradley*, Anglo. 8vo. Edin. 1792.

16. Dissertatio Inauguralis de Cœli Effectibus. Auctore *Jacobo Buchan*, Scoto. 8vo. Edin. 1792.

17 Dissertatio Inauguralis de Rheumatismo acuto. Auctore *Andrea Grieve*, Scoto. 8vo. Edin. 1792.

18. Dissertatio Inauguralis de Hypochondriasi. Auctore *David Corbin Kerr*, Virginiense. 8vo. Edin. 1792.

19. Dissertatio Inauguralis de Variolis. Auctore *Gulielmo Marsden*, Anglo-Britanno. 8vo. Edin. 1792.

20. Dissertatio Inauguralis de Pneumonia.

 Auctore

Auctore *Carolo Merivether*, Virginiense. 8vo. Edin. 1792.

21. Dissertatio Inauguralis de Variolis. Auctore *Roberto Montgomery*, Hiberno. 8vo. Edin. 1792.

22. Dissertatio Inauguralis de Hydrope Anasarca. Auctore *Thoma Pollard Pierce*, Barbadense. 8vo. Edin. 1792.

23. Dissertatio Inauguralis de Angina maligna. Auctore *Georgio Wier*, Scoto. 8vo Edin. 1792.

24. Dissertatio Inauguralis de Alimento. Auctore *Gulielmo Yates*, Anglo. 8vo. Edin. 1792.

25. Dissertatio Inauguralis de Coitu ejusque variis Formis quatenus Medicorum sunt. Auctore *Johanne Paul Gottleib Kircheisen*. 4to. Jena, 1792.

26. Analyse du Systeme absorbant ou lymphatique. Par M. *des Genettes*, D. M. 8vo. Montpellier, 1791.

27. Memoire sur une Maladie de l'Ovaire. Par *Jean Baptiste Ph. R. N. Laumonier*, Chirurgien en chef de l'Hotel Dieu de Rouen. 4to. Rouen, 1790.

28. Avis aux Sages Femmes; par M. *Sacombe*, Medecin

Medecin-Accoucheur, Membre de plusieurs Academies. 8vo. Paris, 1792.

29. Recherches Physico-chymiques. Cahiers I. II. III. 4to. Amsterdam, 1793-4.

30. Memoires de l'Academie Royale des Sciences et Belles Lettres depuis l'Avenement de Frederic Guillaume II. au Trone. 1788 et 1789. Avec l'Histoire pour le même Tems. 4to. Berlin, 1793.

31. Sammlung der Deutschen Abhandlungen welche in der Königlichen Akademie der Wissenschaften zu Berlin vorgelesen worden in den Jahren 1788 und 1789. *i. e.* A Collection of German Essays, read before the Royal Academy of Sciences at Berlin, in the Years 1788 and 1789. 4to. Berlin, 1793.

32. Memoria Chirurgica sul Labbro leporino complicato; di *Giuseppe Sonsis*, R. Assess. Medico, &c. 4to. Cremona, 1793.

33. Pisaura Automorpha e Coreopsis formosa; Piante nuove pubblicate da *Giuseppe Antonio Bonato*, Dott. di Medicina, pubblico Bibliotecario, Ispettore e Soprantendente all' Orto medico dell' Universita di Padova. 4to. Padova, 1793.

INDEX.

### INDEX.

A.

ACID, Vegetable, Solution of Sal Ammoniac in, good effects of, in lacerated Wounds, — 66

Africa, Coast of, Observations on the Diseases most frequent there, — — — 60

Aliment, Dissertation on, —— 226

Ammoniacal Salt, Solution of, in Vinegar, good effects of, in lacerated Wounds, —— —— 66

Anasarca, Dissertation on, —— 226

Aneurism, of the Crural Artery, Case of, — 114

Angina, malignant, Dissertations on, — 225, 226

Animal Electricity, Observations and Experiments relative to, —— —— 161

Antimony, tartarised, recommended as the best Emetic in the Intermittents of Tropical Climates, —— 42

——————, ill effects of nauseating Doses of, during the State of apyrexia in such Cases, *ibid.*

Arsenic, Observations on its Use in Intermittents, 1, 46, 61

———, Effects of, in such Cases, when combined with Peruvian Bark, —— —— 43

———, said to be as powerful and nearly as certain as the Bark, in the Cure of Intermittents; but allowed to be inferior to the Bark in its tonic Effects, — 46

———, Conjecture concerning its specific Action, and that of the Bark, in the Cure of Intermittents, 47

———, Account of irregular Cases of Intermittents in which it fails, —— —— 44

———, Historical Account of its Use in Intermittents, 46

Atmospheres, Electrical, Effects of, on Frogs and other Animals, —— —— 166

B.

Bark, Peruvian, the only Remedy to be relied on in the Intermittents of Tropical Climates threatening immediate Danger, —— —— 44
——————, Conjectures relative to its specific Action in the Cure of Intermittents, —— 47, 143

Bark, Peruvian, Prejudices which ftill prevail againft its Ufe, — — 57
———, different Species of, enumerated, 146
Beddoes, Thomas, Guide for Self-prefervation, 224
Bedford, Arthur, de Angina maligna, — 225
Berlin, Memoirs of the Academy of Sciences at, 227
Bifchoff, Fred. on the Extraction of the Cataract, 223
Blair, Thomas, de Refpiratione, — 225
——, William, of the Extraction of an extraneous Subftance from the Rectum, — — 111
Bonato, G. Anton. Defcription of two new Plants, 227
Bower, Joannes, de Variolis, — 225
Bradifh, Wheaton, de Vifu, — *ibid.*
Bradley, Joannes, de Rheumatifmo acuto, — *ibid.*
Bread, toafted, Inftance of its producing painful Effects in the Rectum, — — 111
Buchan, Jacobus, de Coeli Effectibus, — 225

C.

Cambridge, Catalogue of the Botanic Garden at, 224
Camper, Profeffor, his *Demonftr. Anat. Pathol.* referred to, — — 107, 108
Carey, Matthew, Account of the Yellow Fever at Philadelphia, — — 223
Carter, Henry Yates, on the Effects of Sal Ammoniac and Vinegar on lacerated Wounds, — 66
———, Cafe of a difeafed Kidney, 85
——— Gun-fhot Wound, 91
Cataract, Treatife on the Extraction of, — 223
Caterpillars, not affected by Electricity, — 193
Cinchona, different Species of, enumerated, — 146
Clarke, Robert, Account of a new Key Inftrument, 120
Climate, Differtation on the Effects of, — 225
Coating, Metallic, Advantages of, in Experiments of Animal Electricity, — — 174
———, of two different Metals, Obfervations on the Ufe of, in Experiments of Animal Electricity, 178, 183

D.

Diarrhœa, Effects of Mahogany Wood in Cafes of, 156
Duncan, Dr. Andrew, Junior, his Differtation on the Swietenia Soymida referred to, — 127, 144

E.

Electricity, Animal, Obfervations and Experiments relative to, — — 161

Electricity, Discovery of a new Law in, — 177, 192

———, Estimate of the Quantity of, necessary to produce certain Effects in a Frog, ——— 170

Electrometer, Animal, what so called, — 171

Emetics, good Effects of, in Intermittents, 41, 42

F.

Fever, Epidemic, in Somersetshire, Work relative to, 224

———, Intermittent, of a Tropical Climate, Observations on, ——— ——— 1

———————————, how distinguished from the Remittent, ——— 43

———————————, anomalous Cases of, described, —— —— 44

———————————, See *Antimony*, *Arsenic*, *Bark*, *Emetics*, *Opium*.

———, Intermittent and Remittent, the most frequent Diseases on the Coast of Africa, —— 60

———, Yellow, at Philadelphia, Works relative to, 223

Forster, Thompson, Case of Aneurism of the Crural Artery, —— —— —— 114

Frogs, Effects of Electric Atmospheres on, — 166

G.

Galvani, Lewis, Account of his Discoveries relative to Animal Electricity, ——— —— 161

Genettes, M. des, Analyse du Systeme Lymphatique, 226

Gravel, voided, with Urine, by vomiting, — 215

Grieve, Andreas, de Rheumatismo acuto, —— 225

H.

Hare Lip, Work relative to, —— 227

Head, Case of a Gun-shot Wound of, — 91

Hughes, Francis, on the Effects of Mahogany Wood in Diarrhœa, ——— ——— 156

Hypochondriacal Affection, Dissertation on, — 225

I

Insects, several Species of, not affected by Electricity, 194

Intermittents of a Tropical Climate, Observations on the Use of Arsenic in, —— —— 1

———————————, Use of Opium in, 41

——————————— Emetics in, *ibid.*

———————————, how distinguished from a Remittent Fever, ——— 43

———————————, Circumstances of, in which the Bark alone is to be relied on, —— 44

Intermittents

Intermittents of a Tropical Climate, anomalous Cafes of, defcribed, ——— ——— *ibid.*
Ifchuria, fingular Cafe of, ——— 212
Iffue, fingular Effects of, ——— 109

K.

Kerr, David Corbin, de Hypochondriafi, — 225
Key Inftrument, of a new Conftruction, Account of, 120
———, Obfervations on the Principles on which it acts, ——— ——— 124
Kidney difeafed, Cafe of, ——— 85
———, Appearance of, on Diffection, 89
Kircheifen, I. P. G. Differtatio de Coitu, — 226

L

Lancifi, his Theory of a Cafe in which Urine was voided by vomiting, ——— ——— 221
Laumonier, M. fur une Maladie de l'Ovaire, — 226
Lemery, M. Cafe of a Monk who vomited Urine, 222
Leyden Phial, the fuppofed Analogy of, to fome Phenomena of Animal Electricity, ——— ——— 190
Lizard, Experiment on, ——— 202
Lorimer, Dr. John, Return of the Sick on Board the Eaft-India Company's Ships for the Years 1792 and 1793, 211
Lymphatic Syftem, Work relative to, ——— 226

M.

Mahogany, a new Species of, defcribed, and its Bark recommended as a Subftitute for the Peruvian Bark, 127
———, Wood, Account of its Effects in Diarrhœa, 156
———, of Jamaica, defcribed as more aftringent than that of Honduras, ——— 157
Marangoni, M. Cafe of a Patient who vomited Urine, 220
Marfden, Gulielmus, de Variolis, ——— 225
Melia Azadirachta, the Bark of, recommended as a Subftitute for Peruvian Bark, ——— ——— 154
Merrivether, Carolus, de Pneumonia, — 225
Montgomery, Robertus, de Variolis, ——— 226
Mufcles, Experiments on, 188, 191, 198, 202
———, not *immediately* affected by Electricity, 179, 200
———, voluntary, the only ones affected by weak Currents of Electricity, ——— ——— 197

N.

Naffy, Dr. D. Obfervations on the Epidemic Fever at Philadelphia, ——— ——— 223

Nauclea Daduga, Bark of, its Properties defcribed, 155
Nerves, the only Parts immediately affected by Electricity, — — — 179, 200
———, Effects of certain morbid Alterations of, 96
———, Experiments on, — 181
———, painful Effects of Preffure on, — 101
———, fubcutaneous, a Difeafe of, defcribed, 108
Nott, Dr. John, on the Waters of Pifa and Yverdun, 224

O.

Opium, its Effects in the Intermittents of a tropical Climate, — — — 41
Ovarium, Dropfy of, cured by a fpontaneous vomiting, 221

P.

Pearfon, John, Account of extraordinary Symptoms arifing from certain morbid Affections of the Veins and Nerves, 96
Percival, Dr. his Account of a Dropfy of the Ovarium cured by a fpontaneous vomiting, referred to, — 221
Philadelphia, Works relative to the Yellow Fever at, 223
Pierce, Thomas Pollard, de Hydrope Anafarca, 226
Pifa, Differtation on the Thermal Waters of, — 224
Pneumonia, Differtation on, — 225
Poole, Richard, Account of an Epidemic Fever in Somerfetfhire, — — 224
Pouteau, M. his Pofthumous Works quoted, 103

Q.

Quadrupeds, Experiments on, — 181, 187

R.

Recherches Phyfico-chymiques, — 227
Remittent Fever, *See Fever.*
Refpiration, Differtation on, — 225
Rheumatifm, acute, Differtation on, — 225
Roxburgh, Dr. W. Account of a new Species of Swietenia, — — 127
Rufh, Dr. Benj. of the Bilious Remitting Yellow Fever, 223

S.

Sacombe, M. Avis aux Sages Femmes — 226
Sal Ammoniac.—*See* Ammoniacal Salt.
Senter, Dr. Ifaac, of a fingular Cafe of Ifchuria 212
Sibthorp, Joannes, Flora Oxonienfis — 224
Sierra Leone, Account of the Weather at, — 2
Small Pox, Differtations on, — 225, 226
Sonfis, G. his Work relative to the Hare Lip, 227
Stimuli, Work relative to the Effects of, — 223

Swietenia, Accounts of two new Species of, 127, 153

T.

Teeth, Obſervations on the Extraction of, by the Key Inſtrument, —— —— 121

Tongue, human, Experiments on, —— 208

——, of Quadrupeds, Experiments on, 209, 210

Typhus, Work relative to the Nature and Cure of, 223

V.

Vinegar, Solution of Sal Ammoniac in, Effects of, on lacerated Wounds, —— —— 66

Viſion, Diſſertation on, —— 225

Volta, Alexander, Tranſlation of his Letters on Animal Electricity, —— —— 161

Urine, Caſes in which it has been voided by vomiting, 212, 220, 222

W.

Wallurſe, a Tree ſo called by the Hindoos, deſcribed, 154

Wier, Georgius, de Angina maligna, — 226

Winterbottom, Dr. T. M. on the Uſe of Arſenic in Intermittents, —— —— 1

——————, Account of the Weather at Sierra Leone, —— —— 2

Worms, not affected by Electricity, —— 194

Wood, Dr. J. on the Application and Abſtraction of Stimuli, —— —— —— 223

Y.

Yates, Gulielmus, de Alimento, —— 226
Yellow Fever, Works relative to it, —— 223
Yverdun, Work relative to the ſulphureous Water of, 224

END OF THE SIXTH VOLUME.

